# The helioscope effect: A new framework for evaluating trauma-related memory processing in psychedelic experiences

**DOI:** 10.1177/02698811251397306

**Published:** 2025-12-31

**Authors:** Vincent J. Diehl, Abigail E. Calder, Gregor Hasler

**Affiliations:** 1 Molecular Psychiatry Lab, Faculty of Science and Medicine, University of Freiburg, Villars-sur-Glâne, Switzerland; 2 Lake Lucerne Institute, Vitznau, Switzerland; 3 Freiburg Mental Health Network, Villars-sur-Glâne, Switzerland

**Keywords:** psychotherapy, resilience, exposure, safety

## Abstract

**Background:**

Existing tools assess psychedelic experiences, but none specifically measure altered processing of traumatic memories—a key mechanism in trauma-focused therapies and psychotherapy in general. The helioscope effect describes how psychedelics like psilocybin and 3,4-methylenedioxymethamphetamine (MDMA) enable revisiting challenging or traumatic experiences while remaining protected from re-actualization of trauma symptoms. This study introduces and evaluates the Helioscope Questionnaire, a novel scale for assessing memory-related processing during psychedelic experiences.

**Method:**

A cross-sectional, Internet-based survey was administered to 468 individuals (mean age = 32.9; 66.7% male) with self-reported psychedelic/MDMA use.

**Results:**

The final Helioscope Questionnaire comprised 21 items across 3 factors: *protection*, *exposure*, and *avoidant*-*distress*. A composite Helioscope Score (HS) was derived from protection and exposure subscales. Convergent validity was demonstrated through strong correlations with the Psychological Insight Questionnaire. Discriminant validity was evidenced by moderate associations with the Mystical Experience Questionnaire and a lack of significant correlations with the Challenging Experience Questionnaire. Predictive validity was supported by the HS predicting positive changes in mood and attitude on the Persisting Effects Questionnaire, whereas *avoidant-distress* predicted negative changes. The scale also demonstrated incremental validity by providing explanatory power beyond established psychedelic effect measures. Additionally, the presence of a trip sitter was associated with stronger HS scores, and MDMA use was linked to reduced avoidant distress.

**Conclusions:**

The Helioscope Questionnaire offers a novel, psychometrically robust tool for assessing therapeutic mechanisms of psychedelic experiences, particularly in relation to processing of difficult memories. Further research in clinical populations is warranted to evaluate its utility in predicting treatment outcomes.

## Introduction

The involuntary re-experiencing of traumatic events is a hallmark of posttraumatic stress disorder (PTSD; American Psychiatric Association, 2022), imposing a significant burden on those affected. Clinical guidelines recommend trauma-focused psychological therapies, such as cognitive processing therapy and prolonged exposure therapy, as standard treatments for PTSD (Martin et al., 2021). These interventions help individuals confront traumatic memories in a structured and controlled manner, fostering the potential for resolution (McLean et al., 2022; Oren and Solomon, 2012). However, they can also be highly distressing, which contributes to their dropout rates of 18%–30% (Eftekhari et al., 2020; Lewis et al., 2020). Furthermore, third-wave cognitive behavioral therapies, such as mindfulness-based interventions, have been linked to negative outcomes, particularly when they expose individuals to distressing levels of traumatic content (Britton et al., 2021). In addition to psychotherapy, selective serotonin reuptake inhibitors are commonly prescribed as first-line pharmacological treatments for trauma-related disorders (Martin et al., 2021). However, their effectiveness remains limited, and they are often associated with adverse effects, including emotional numbing (Williams et al., 2022).

A novel therapeutic approach for PTSD involves the use of the connectogen 3,4-methylenedioxymethamphetamine (MDMA) which, when combined with psychotherapy (MDMA-AP), has shown significant efficacy in reducing PTSD symptoms (Mitchell et al., 2021; Mithoefer, 2015; Mithoefer et al., 2018; Stocker and Liechti, 2024). Trials have shown that MDMA-AP may be more effective than previous treatments (Amoroso and Workman, 2016; Smith et al., 2022) and may be more acceptable to many patients than other forms of trauma treatments (Krieger, 2018; Lee and LaFreniere, 2023; Mott et al., 2014; Perrone, 2024; Vogel et al., 2020). MDMA’s efficacy may be partly attributed to its ability to facilitate safer and more constructive engagement with traumatic memories. The *Manual for MDMA-Assisted Psychotherapy in the Treatment of Posttraumatic Stress Disorder* highlights that MDMA-AP may allow individuals to effectively confront traumatic memories (Mithoefer, 2015). Furthermore, MDMA appears to enhance the vividness and positivity of positive memories while reducing the overwhelming nature of negative memories, without diminishing their emotional quality or detail (Carhart-Harris et al., 2014). Notably, initial human research shows that MDMA has the potential to enhance fear extinction retention, suggesting one possible mechanism for its therapeutic effects in trauma treatments (Vizeli et al., 2022). Similar to other psychedelics, the effectiveness of MDMA-AP likely depends on factors such as the therapeutic setting and the individual’s mindset (Carbonaro et al., 2016; Carhart-Harris et al., 2018; Haijen et al., 2018; Holze et al., 2021).

MDMA’s protective effect in people processing traumatic memories has been illustrated with the “helioscope” metaphor (Hasler, 2022b). Helioscopes enable detailed observation of the Sun by blocking harmful radiation; likewise, MDMA can act as a protective filter which softens the intensity of distressing memories to allow for safer, more detailed processing and integration. Though perhaps most often observed in MDMA-AP, the helioscope effect may also occur with classic psychedelics. Previous research on psilocybin-assisted therapy (psilocybin being the active compound in “magic mushrooms”), and ayahuasca ceremonies (a traditional Amazonian plant brew) showed that re-experiencing and re-evaluating traumatic memories during the psychedelic session can positively influence depressive symptoms (Watts et al., 2017), foster abstinence from an addictive substance (Noorani et al., 2018) and significantly reduce neuroticism (Weiss et al., 2023).

The helioscope effect encompasses two key dimensions of processing traumatic, stressful, and otherwise adverse experiences (Hasler, 2022b). First, someone experiencing the helioscope effect is engaging with difficult or traumatic experiences, known as trauma exposure (Rothbaum and Schwartz, 2002). Instead of this being overwhelming, as it might normally be, there is a perception of relative safety and reduced emotional overwhelm. Together, trauma exposure and protection from emotional overwhelm address well-documented challenges in trauma-focused therapies (Foa, 2011; Lewis et al., 2020) and align with recent findings on the role of psychedelics in promoting relaxation and emotional regulation during psychedelic-assisted therapy (PAT) (Calder et al., 2024).

Although numerous questionnaires assess the subjective effects of psychedelics, no existing instrument specifically evaluates the mechanisms underlying traumatic memory processing in PAT. To investigate altered memory processing under MDMA and classic psychedelics, the last author (G.H.) developed the Helioscope Questionnaire (HQ), a specialized instrument designed to assess whether people experience the helioscope effect during a psychedelic experience. The HQ is designed to capture the two essential components of the helioscope effect, namely exposure and protection. Additionally, the HQ includes questions capturing avoidance of and distress at difficult experiences, which are common issues in PTSD which can emerge in classical exposure therapies and even result in a symptom exacerbation (Lewis et al., 2020). Given the central role of trauma processing in MDMA-assisted psychotherapy in particular, the HQ offers a novel approach to understanding how PAT achieves similar outcomes and lower dropout rates compared to conventional exposure therapies (Amoroso and Workman, 2016).

This study had two primary aims. First, we investigated the HQ’s validity and psychometric properties using a cross-sectional, international survey of people who had recently used MDMA or classic psychedelics. We hypothesized that the HQ would break down into three distinct and relatively independent factors, namely trauma exposure, protection, and avoidance/distress. We assessed convergent and discriminant validity using measures of psychological insight, mystical experiences, and challenging experiences, which are established predictors of long-term changes following psychedelic use (Carbonaro et al., 2016; Davis et al., 2021; Ko et al., 2022). Additionally, we examined whether a stronger helioscope effect predicted positive persisting effects and provided incremental explanatory power beyond these existing measures. Following this, our second aim was to evaluate potential predictors of the helioscope effect, including drug class (MDMA vs. classic psychedelics) and extrapharmacological factors.

## Methods

### Participants

Study participants were recruited through online forums dedicated to the discussion of psychedelics, word of mouth, and advertising via academic institutions and conferences focusing on the topic. They were invited to participate in a study on “the acute effects of psychedelics on memory processing.”

#### Inclusion and exclusion criteria

To be eligible for the study, participants needed to be at least 18 years of age and fluent in English or German. Furthermore, participants had to have consumed a psychedelic drug that resulted in a discernible psychedelic experience within the past 12 months. The 12-month recall period was chosen to balance capturing recent psychedelic experiences while minimizing recall bias. For this study, we defined psychedelic drugs as lysergic acid diethylamide (LSD) or its analogs (e.g., 1P-LSD), psilocybin or psilocybin-containing mushrooms (commonly referred to as magic mushrooms or magic truffles), dimethyltryptamine, 5-methoxy-N,N-dimethyltryptamine, Ayahuasca, Mescaline or Peyote, 2C-B, or MDMA. Notably, we only recruited participants who were not under the influence of any other drugs, psychiatric medications, or alcohol during their reported psychedelic experience. Finally, participants were excluded if they failed attention checks, which involved responding to specific test items with predetermined answers (e.g., “Please answer ‘Moderately’ to this question”).

### Study procedure

This study was an anonymous retrospective survey. Advertisements led potential respondents to the web-based assessment, which was conducted using the secure REDCap platform (Vanderbilt University, Nashville, Tennessee, USA). All participants gave their informed consent to participate in the study. The study was approved by the Internal Review Board of the Department of Psychology at the University of Fribourg, Switzerland (Ref-No.: 2023-862). As an incentive, participants were able to take part in a randomized raffle for one of five 50 Euro Amazon gift cards after successfully completing the survey.

### Development of the Helioscope Questionnaire

The HQ was developed to assess the extent to which participants were able to confront and process difficult or traumatic memories, emotions, thoughts, and stressful topics during their psychedelic experience. The last author (G.H.) developed the HQ, based on existing literature describing trauma processing in various therapy modalities, including PAT, as well as his clinical and naturalistic experience using psychedelics in psychotherapy based on the Swiss limited medical use program (Liechti et al., 2025). Great care was taken to incorporate diverse wording and descriptions of the included phenomena to accommodate different theoretical perspectives and enhance overall comprehension. Questions probed distress levels, coping ability, feelings of security and relaxation, memory recurrence, gained insights, and clarity of recalled experiences. The initial pool of questions consisted of 24 items. The questionnaire was then refined in one iterative consultation with Swiss psychiatrists who were either experts in psycho-traumatology and/or conducted psychedelic-assisted psychotherapy under the country’s limited medical-use program (Calder and Hasler, 2023; Gasser, 2017). Each psychiatrist was asked to rate the items for clarity and clinical relevance, flag redundancy, and suggest additional facets of the proposed helioscope effect. These consultations led to a rephrasing of several items and the addition of an item assessing potential physical relaxation. The final HQ comprised 25 items (Table S1) rated on a 5-point Likert scale from “completely agree” to “completely disagree.” Originally developed in German, the scale was translated into English by two clinical researchers and one other translator, including one German and two English native speakers. To ensure the accuracy and fidelity of the translation, it was then translated back into German by two German-speaking researchers, and discrepancies were resolved by discussion (Beaton et al., 2000). Both language versions were used for validation in this study.

### Study measures

#### Identification of a single psychedelic experience within the last 12 months

Participants were asked to report on a psychedelic experience they had had in the past 12 months. If participants had more than one such experience within this period, they were asked to refer to the one in which the “occurrence of past memories and experiences was most prevalent.”

##### Demographics

This survey included questions about participants’ age, sex, marital status, country of residence, ethnicity, and educational level as they were at the time of their psychedelic experience.

##### Drug, set, and setting

We asked about participants’ mindset and environment (“set and setting”) during their psychedelic experience. The pool of questions was developed in our research group based on literature mentioning the influence of these dimensions on the psychedelic experience (Golden et al., 2022). We asked whether the substance was consumed individually or in a group, their feelings toward their social environment, and their perceptions of the overall environment. Questions varied from asking about the physical surroundings (“The setting was. . .”) with multiple choice answers (Mostly indoors; Mostly outdoors in nature; Mostly outdoors, but not in nature; Somewhere else, please specify) to questions about a perceived feeling (How safe did you feel in this setting?) which could be answered using a Visual Analog Scale ranging from “Not safe at all” to “Completely safe.” Additionally, we asked about the drug and the dose taken which exerts a strong influence on the quality of the experience (Griffiths et al., 2011). Here, we also asked about how the drug was consumed, where it was acquired from, and when it was taken. Participants were also asked about their intentions for taking the psychedelic substance which could be chosen from a list of 14 items. For the analysis we grouped them by content into social, conformity, coping, enhancement, and expansion as previously indicated by a systematic review on motives for the use of psychedelics (Basedow and Kuitunen-Paul, 2022) (Table S2).

#### Acute psychedelic experience

To assess the broad range of acute effects of the psychedelic experience we included the following scales.

##### Helioscope Questionnaire

Participants rated their agreement with statements on the HQ using a five-point scale (1 = “Completely disagree” to 5 = “Completely agree”), with an option for “Not applicable/cannot say” (NA). Additionally, participants provided feedback on the relevance of the HQ questions to their experience “Were the questions in this questionnaire relevant to your psychedelic experience?” (0 = No, not at all; 100 = Yes, extremely relevant), as well as perceived stress (“Was answering the questionnaire stressful for you?” (0 = No, not at all; 100 = Yes, extremely stressful) and pleasure associated with completing the questionnaire (“Was answering this questionnaire enjoyable for you?” (0 = No, not at all; 100 = Yes, extremely pleasant)), and whether a singular memory or experience came to mind during their session. The cut-off value for the presence of the helioscope effect was set at a total score reflecting an average of over “Neutral” (value of 3). Additionally, a higher score of 1.5 times that value was chosen to reflect a strong helioscope effect.

##### Mystical experience

The Mystical Experience Questionnaire (MEQ) was employed to evaluate the extent of subjective mystical experiences reported by participants (Barrett et al., 2015). The MEQ comprises 30 items, each rated on a 6-point scale ranging from “None; not at all” to “Extreme (more than ever before in my life).” The total MEQ score was calculated for use in the analysis. Given that the MEQ primarily assesses mystical experiences, while the HQ focuses on the processing of distressing memories, we anticipated a low-to-moderate correlation (*r* = 0.1–0.5) between these measures, reflecting their assessment of related but distinct constructs.

##### Challenging experience

The Challenging Experiences Questionnaire (CEQ), developed by (Barrett et al., 2016), was utilized to assess the extent of challenging qualities during a psychedelic experience. The CEQ consists of 26 items, each describing psychological or physical effects that can be perceived as challenging under the influence of psychedelics. Respondents rated the intensity of their experiences on a 6-point scale ranging from 1 (“None, not at all”) to 6 (“Extreme, more than ever before in my life”). We hypothesized that items of the HQ assessing avoidance and distress would show at least moderate correlation with the total CEQ score (*r* > 0.3), as challenging re-experiencing of distressing memories is conceptually aligned with the CEQ’s measurement of burdensome psychological effects. The total score, calculated as described in the original publication on this scale (Barrett et al., 2016), was used for analysis.

##### Psychological insight

The Psychological Insight Questionnaire (PIQ; Davis et al., 2021) was utilized to measure the degree of insights experienced by participants during their psychedelic session. The PIQ consists of 28 items, each rated on a six-point scale from 0 (“No; not at all”) to 5 (“Extremely (more than ever before in my life”). Responses were summed to produce a total PIQ score, with higher scores indicating a greater degree of insight experienced. We hypothesized that the PIQ and the HQ would correlate moderately to highly (*r* > 0.3), as they address related concepts. Insight reflects a broader perception of understanding or realization, and the memory processing described in the HQ could be viewed as a process possibly leading to insight.

##### Persisting effects

The Persistent Effects Questionnaire (PEQ; Griffiths et al., 2006) was employed to evaluate the enduring impact of participants’ psychedelic experiences. Four subscales were used: Positive Attitude about Life or Self, Negative Attitude about Life or Self, Positive Mood Changes, and Negative Mood Changes. These subscales of the PEQ include 42 items asking participants to rate how the experience influenced various aspects of their lives on a 6-point scale (0 = “Not at all” to 6 = “Extreme, more than considered humanly possible”). For each subscale, a total score was calculated. These were further aggregated into two composite scales: one reflecting positive changes in mood and attitudes and the other negative change in mood and attitudes. We hypothesized that the helioscope effect would significantly predict positive changes and that the negative items of the HQ significantly predict negative changes assessed with the PEQ.

### Data analysis

All analyses were conducted using the statistical software R (Version 2023.09.1+494) (Posit PBC, Boston, MA). Prior to analysis, the dataset was screened for unlikely survey completion times. No participants with unusually short completion times were identified.

Before analyzing the factor structure of the HQ, missing values resulting from “Not applicable/cannot say” (NA) responses were imputed using the mice package (v3.16.0: Buuren and Groothuis-Oudshoorn, 2011) with the random forest method, a robust approach for handling questionnaire data. This approach is based on recommendations on how to handle missing data (Schreiber, 2021; Watkins, 2018). Additionally, participants with more than 2/3 of items indicating NA were excluded from the analysis. Detailed methods for assessing equivalence between the imputed and complete-case datasets, as well as between the English and German versions, are described in the Supplemental Material.

Before factor analysis, we examined polychoric correlations between items to determine whether any should be removed (items correlating >0.9 with any other item, as well as items correlating <0.3 with all other items, are candidates for removal (Yong and Pearce, 2013)). Next, parallel analysis and eigenvalues were calculated to determine the number of factors to retain. Exploratory factor analysis (EFA) was subsequently conducted using a polychoric correlation matrix and the weighted least squares method, which is well suited for nonnormally distributed ordinal data (Xia and Yang, 2019). Promax rotation was applied because correlations among factors were anticipated. Items with factor loadings <0.3 on all factors or high cross-loadings (with differences ⩽0.1 across factors) were excluded to ensure the scale’s clarity and relevance, following established guidelines in psychometrics (Kline, 2014; Watkins, 2018; Yong and Pearce, 2013). This was iteratively done until the factor structure, and all loadings were unambiguous. The EFA was performed using the *psych* package (v2.3.6, Revelle, 2024).

To assess convergent and discriminant validity, Spearman’s correlations were calculated between the HQ and the MEQ, CEQ, and PIQ. The Benjamin–Hochberg correction was applied to account for multiple comparisons and control the false discovery rate. Next, predictive and incremental validity were evaluated through multiple linear regressions or, when appropriate, robust regressions conducted using the *MASS* package (v7.3-60: Venables and Ripley, 2013). In the first model, a linear regression was conducted to examine the HQ score as a predictor of positive changes in mood and attitudes as measured by the PEQ. This was followed by two multiple regression models: the first included the MEQ and PIQ scores as predictors of positive changes, while the second added the HQ score to assess its incremental contribution. A similar analysis was performed to evaluate whether the negative HQ subscale predicted negative changes on the PEQ. Here, we assessed whether the negative HQ subscale improved the predictive power of models already containing the CEQ scores. Statistical difference between the models was tested using analysis of variance for linear models and with Linear Regression Model (LRM) in the case of robust linear models.

Finally, after assessing the HQ’s validity and factor structure, the influence of drug type and extrapharmacological factors on HQ scores were examined using multiple regressions. The dataset consisted of self-reported responses assessing contextual variables and subjective experiences associated with psychedelic use. Variables included experiential ratings (e.g., intensity, positivity), setting characteristics (e.g., sitter presence, ceremonial or therapeutic context), and participant intentions for use (e.g., coping, enhancement, expansion). Categorical variables were recoded into factors, and binary dummy variables were created where applicable. Setting types were similarly structured into three binary variables: recreational (reference group), ceremonial, and therapeutic. A regularized regression framework was used to finalize predictor sets. For the helioscope effect, least absolute shrinkage and selection operator (LASSO) regression was employed to identify variables contributing to predictive accuracy. For the avoidant distress factor (ADF), given a larger number of retained predictors from LASSO, ridge regression was used to manage multicollinearity while preserving all coefficients. Subsequently, robust linear regression models were fitted using the rlm() function from the MASS package to account for violations of standard linear model assumptions (i.e., nonnormal residuals and heteroscedasticity, as confirmed via Shapiro–Wilk, Breusch–Pagan, and Durbin–Watson tests; v7.3-60: Venables and Ripley, 2013). To obtain reliable estimates and inferential statistics, bootstrapping with 1000 resamples was conducted for each robust regression model (Canty, 2002). Bias-corrected and accelerated (BCa) confidence intervals were calculated for each coefficient. Pseudo *p*-values were computed as two times the smaller proportion of bootstrap samples yielding coefficients ⩽0 or ⩾0. Coefficients with *p* < 0.05 were interpreted as statistically significant.

## Results

### Sociodemographic characteristics

Sample demographics are summarized in Table 1. A total of 1333 participants provided informed consent and passed the screening. Of these, 351 did not finish the survey, 301 were excluded for failing attention checks, and 197 were excluded because the answers seem to have been produced by bots. We came to this conclusion because all 197 answers were completed in a time span of a few minutes and showed highly similar patterns. Additionally, excluded 16 participants due to insufficient data on the HQ (>2/3 “Not Applicable”). The final sample included 468 participants with 379 English-speaking and 89 German-speaking individuals.

**Table 1.** Sample characteristics (N=468)

Characteristic	Value
Age	
Mean (SD)	32.9 (12.75)
Sex (%)	
Male	312 (66.7%)
Female	152 (32.4%)
Other	4 (0.9%)
Education	
College degree	288 (61.5%)
Doctoral degree	41 (8.8%)
Ethnicity (multiple choices possible)	
African	10
Asian	23
Hispanic	29
European/Caucasian	398
Native	6
Mideast	23
Other	22
Psychedelic substance	*N* (*Mean Dose; SD*)
Psilocybin containing mushroom dried or other	125 (*3.54g; 3.15*)
Psilocybin containing mushroom wet	18 (*16.59g; 14.62*)
Psilocybin other form	18
LSD or an LSD analog	152 (*210µg; 175.16*)
MDMA	51 (*175mg; 81.84*)
Ayahuasca	30 (*160ml; 261.33 or 1.9 cups; 0.64*)
DMT	15 *(33mg; 25.97)*
5-MeO-DMT	14 *(44mg; 30.57)*
2CB	33 (*25mg; 11.04*)
Mescaline	12 (*307ml; 83.76* or *45cm; 33.66*)

5-MeO-DMT: 5-Methoxy-N,N-dimethyltryptamin; 2CB: 4-Brom-2,5-dimethoxyphenylethylamin; MDMA: 3,4-methylenedioxymethamphetamine; LSD: Lysergic acid diethylamide.

### Acute psychedelic experience

**Figure 1.**
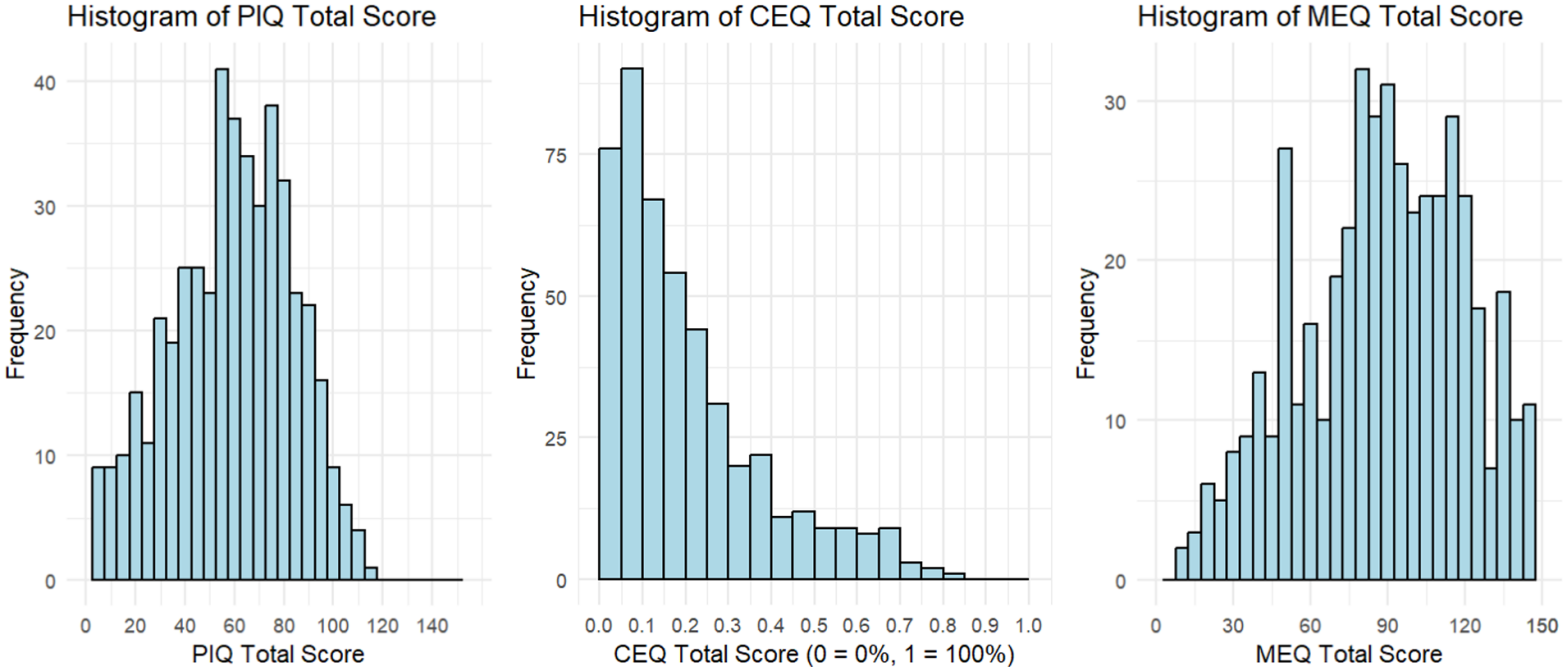
Distributions of total scores for the CEQ, MEQ, and PIQ. The x-axis shows the full range of possible scores for each scale. Data represent responses from *N* = 468 participants. CEQ: Challenging Experience Questionnaire; MEQ: Mystical Experience Questionnaire; PIQ: Psychological Insight Questionnaire.

When asked to choose a recent psychedelic experience, most respondents reported on an experience with psilocybin-containing mushrooms, LSD and its analogues, or MDMA, with doses typically in the medium-to-high range (mean dose for dried mushrooms = 3.5 g; mean dose for LSD = 210 µg, mean dose for MDMA = 175.4 mg). Other substances used were reported at comparably high dosages (Table 1). Participants rated their experiences as subjectively intense (Mean = 78.5, SD = 14.6, range = 20–100/100). The distribution of subjective effects suggests that participants had moderately strong, relatively positive psychedelic experiences (Figure 1).

Most participants experienced their trips indoors (*n* = 328, 70%) and, in small groups (*n* = 204, 43.6%) and most were accompanied by a trip sitter at least part of the time (*n* = 296, 63.2%). Among those with a sitter, the reported comfort level was high (Mean = 91.45, SD = 13.13) on a scale ranging from 0 (*not at all comfortable*) to 100 (*extremely comfortable*). Likewise, feelings of comfort and safety during the experience were also rated highly (Mean = 85.63, SD = 18.68; Mean = 89.91, SD = 15.14, respectively).

The most common intentions were introspection (e.g., “to help learn about myself”; *n* = 313) and gaining new perspectives (e.g., “to understand things differently”; *n* = 303). Only four participants indicated having no particular intention for their trip (multiple selections were possible). Participants generally reported that their experiences aligned well with their expectations (Mean = 78.30, SD = 24.89), also rated on the 0–100 scale.

### Exploratory factor analysis

**Figure 2.**
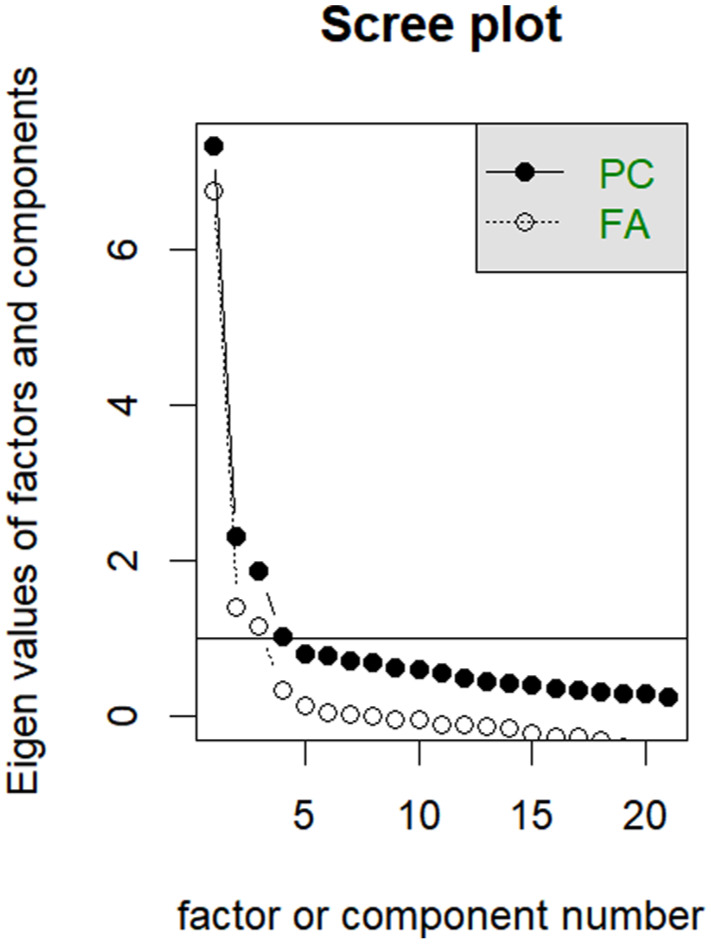
Scree plot displaying eigenvalues of extracted components. Both PC and FA are shown to assess dimensionality and component retention for the Helioscope Questionnaire. PC: Principal components; FA: Factor analysis.

No items were excluded before EFA based on high or low correlations (Figure S1), the Kaiser–Meyer–Olkin test indicated excellent sampling adequacy (Measure of Sampling Adequacy [MSA] = 0.92). Item-level MSAs all exceeded the recommended minimum of 0.50; therefore, no items were flagged for removal. Bartlett’s test of sphericity was significant, χ^2^(300) = 5166.73, *p* < 0.001, rejecting the null hypothesis that the correlation matrix is an identity matrix. Kaiser’s criterion and the scree plot suggested the presence of three factors with eigenvalues >1 (Figure 2). Parallel factor analysis indicated five factors, but analyses with four and five factors resulted in overfitting, as factors 4 and 5 contained only one item each and contributed minimally to explained variance (Kline, 2014; Watkins, 2018; Yong and Pearce, 2013). Thus, a three-factor model was deemed optimal. Following initial EFA, Items 1, 11, 16, and 22 were excluded due to low differential loadings (<0.1) across all factors. These exclusions were supported by content evaluation, identifying the items as either ambiguous or overly general.

The final EFA, conducted with 21 items, yielded 3 factors (Table 2). The first factor, which we termed the Exposure Effect, comprised 9 items (2, 6, 7, 9, 10, 12, 14, 18, and 19) related to the emergence of difficult memories or topics as well as of confrontation with avoided topics. The second factor, Protection Effect, included 8 items (1, 3, 5, 8, 16, 17, and 21) related to the sense of safety during the experience. The third factor, ADF, contained 4 items (4, 13, 15, and 20) assessing negative impacts on processing difficult experiences. Collectively, these three factors explained 47% of the variance, with exposure accounting for 20%, protection 18%, and avoidant distress for 9%. The model demonstrated excellent fit indices (Fit based on diagonal values = 0.99, root mean square of residuals = 0.04) with an average item complexity of 1.3, indicating good model simplicity. We analyzed the imputed data for both languages combined and provide results of comparability checks between imputed and nonimputed datasets and across languages in the Supplemental Material (Table S3 and S4).

**Table 2.** Factor analysis

Item	Exposure	Protection	Avoidance	Com.
1. During the session, I could endure traumatic or difficult memories, feelings, or thoughts better than usual.	0.24	**0.53**	0.19	1.7
2. The session helped me to let go of difficult experiences or memories, or to accept them better.	**0.54**	0.25	0.15	1.6
3. During the session, I experienced a sense of security and safety which allowed me to engage with stressful topics.	0.04	**0.67**	0.20	1.2
4. During the session, I avoided difficult feelings or memories more than usual.	−0.15	0.32	**0.73**	1.5
5. Memories, sensations, or thoughts which normally cause unpleasant feelings were easier to endure during the session.	0.04	**0.66**	−0.13	1.1
6. The session helped me address crucial life questions that I otherwise avoid.	**0.67**	−0.03	−0.01	1.0
7. The session gave me access to memories or feelings that were not fully conscious before.	**0.57**	−0.14	0.08	1.2
8. During the session, I was surrounded by a protective armor that kept memories and feelings from hurting me.	−0.15	**0.79**	0.18	1.2
9. During the session, I saw difficult situations and experiences from my past more clearly and with more detail.	**0.71**	−0.04	0.08	1.0
10. The session allowed me to engage with distressing memories, emotions, or thoughts that I normally cannot bear.	**0.61**	0.15	0.07	1.1
11. During the session I could bear difficult physical sensations better than usual.	0.12	**0.47**	0.06	1.2
12. Because the session allowed me to confront the challenging and distressing aspects of my life, I was able to process them better.	**0.74**	0.07	0.10	1.1
13. During the session, I experienced stress that made it more difficult for me to engage with psychologically important topics.	0.19	−0.18	**0.57**	1.4
14. After the session, I was better able to withstand distressing memories, thoughts, or feelings.	**0.52**	0.19	0.01	1.3
15. During the session I was less able to withstand difficult emotions or memories than usual.	0.18	−0.14	**0.48**	1.5
16. During the session I felt that I possessed a protective filter which allowed me to withstand difficult emotions or memories.	−0.06	**0.76**	0.19	1.1
17. During the session I was able to confront negative memories without being overwhelmed by them.	0.32	**0.51**	−0.14	1.8
18. During the session I felt like I was being guided to those traumatic experiences which were ready to be psychologically processed.	**0.61**	0.11	0.02	1.1
19. The session helped me to better understand my history of trauma.	**0.67**	0.05	0.01	1.0
20. During the session, I avoided difficult issues and experiences even more than usual.	−0.16	0.27	**0.81**	1.3
21. The relaxation I felt during the session helped me deal with difficult life events.	0.14	**0.56**	0.07	1.2

Com: Communalities. Bold values indicate the highest loading in each row.

Exposure and protection demonstrated excellent reliability (ω = 0.91, *Cronbach’s* α = 0.88; ω = 0.90, *Cronbach’s* α = 0.87, respectively). Avoidant distress had slightly lower but still acceptable reliability (ω = 0.78, *Cronbach’s* α = 0.72). The total scale exhibited excellent internal reliability (ω = 0.94, *Cronbach’s* α = 0.91) (McDonald, 2013). Correlations between subscales indicated a moderate positive relationship between exposure and protection (*r* = 0.58, *p* < 0.001), while both exposure and protection correlated negatively with avoidant distress (*r* = −0.14, *p* = 0.002 and *r* = −0.21, *p* < 0.001, respectively).

Based on these results, a total score for the helioscope effect was derived by summing the exposure and protection subscale scores, which together represent encounters with and protection from distressing memories, thoughts, and emotions. Thus, a higher HS indicates greater facilitation of difficult memory processing, with a maximum score of 85. Additionally, when using the avoidant distress as a standalone measure, higher scores reflect stronger avoidance of difficult experiences and intensified distress. The full questionnaire and scoring manual can be found in the Supplemental Material (see Figures S2 and S3).

### Helioscope scores and experience to complete the questionnaire

**Figure 3.**
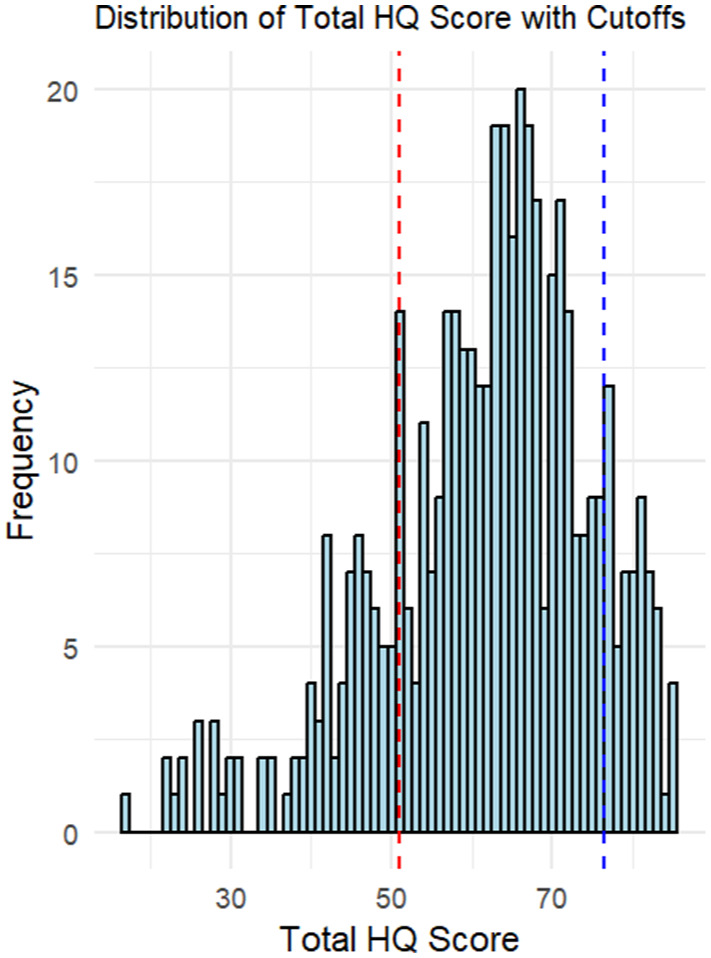
Histogram showing the distribution of helioscope total scores. The red dashed line represents the cutoff for effect presence, while the blue dashed line represents the threshold for a strong effect presence.

In our sample, the median of the HS was 64 (Range = 17–85/85). Participants generally rated the questionnaire items as relevant to their psychedelic experiences (Median = 75, Range = 0–100/100), based on the single-item measure. Completing the questionnaire appeared to be minimally stressful for most participants (Median = 1, *Range* = 0–88/100), as assessed by the single item. Interestingly, participants reported a positive experience completing the questionnaire (Median = 71, Range = 0–100). A total of 255 of the 468 participants endorsed having confronted a particular memory or experience from their past during the psychedelic experience. After calculating the cut-off values the data showed that 78.8% of participants scored more than neutral on the HS (Figure 3), while only 10.5% scored more than neutral on the ADF.

### Convergent validity

**Figure 4.**
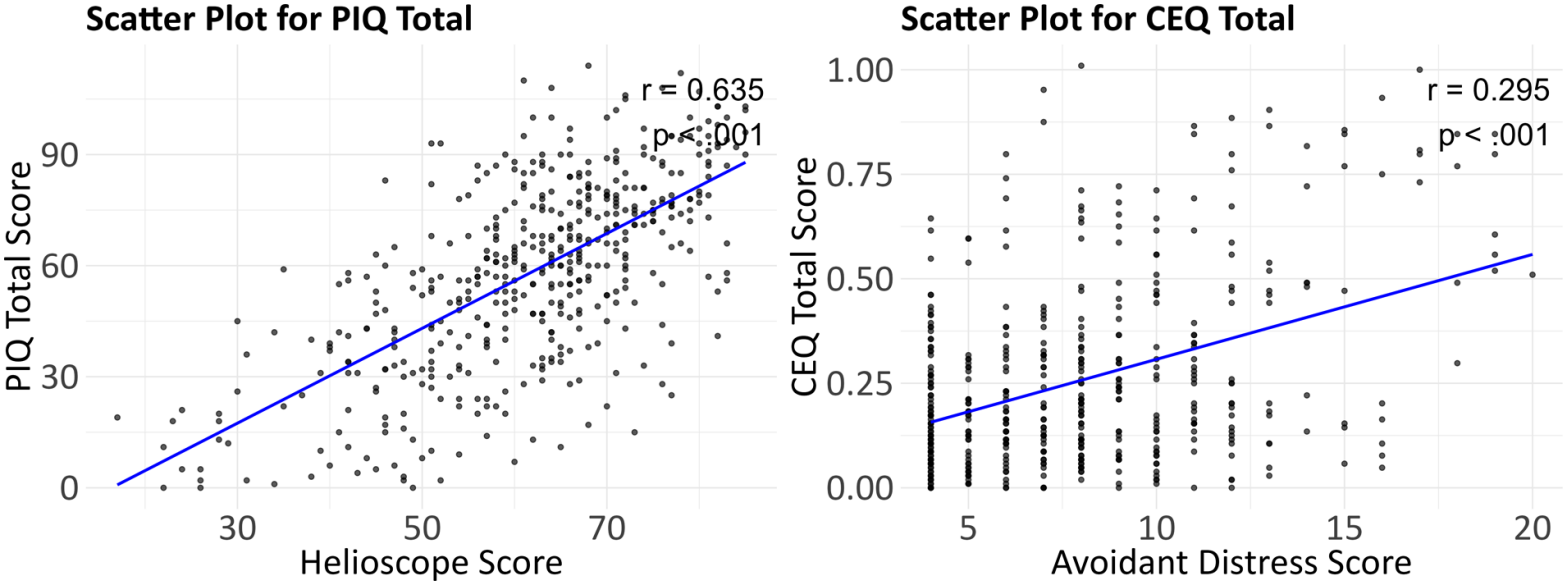
Scatterplots of Spearman’s correlations between PIQ with helioscope score and CEQ total scores with avoidant distress score. Left panel: Moderate positive association between helioscope score and PIQ total score. Right panel: Moderate positive correlation between avoidant distress and CEQ total score. CEQ: Challenging Experience Questionnaire; PIQ: Psychological Insight Questionnaire.

The convergent validity of the HQ was examined by assessing correlations with the PIQ. As hypothesized, a strong positive correlation was observed between the HS and the PIQ (Figure 4). We also investigated the correlation between avoidant distress and the CEQ. Consistent with our hypothesis, the ADF demonstrated a moderate positive correlation with the CEQ total score (Figure 4).

### Discriminant validity

**Figure 5.**
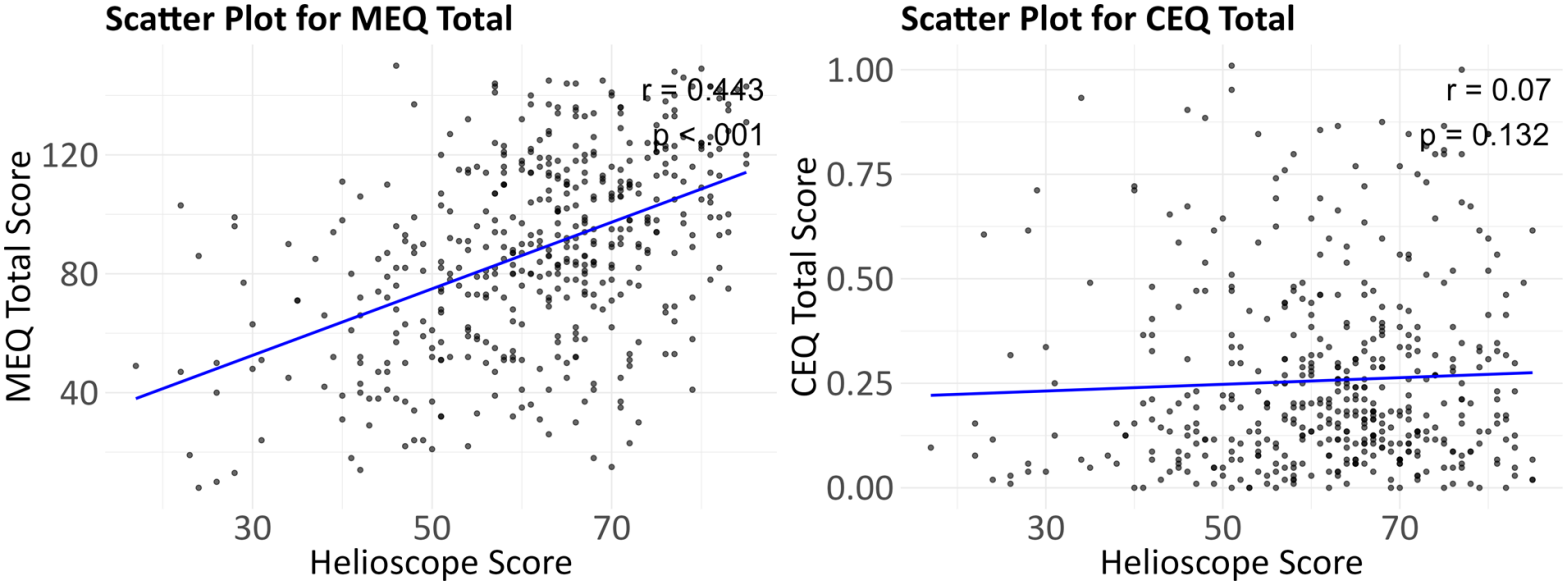
Scatterplots of Spearman’s correlations between MEQ and CEQ total scores with helioscope score. Left panel: Moderate positive correlation between helioscope score and MEQ total score. Right panel: Weak nonsignificant correlation between helioscope score and CEQ total score. CEQ: Challenging Experience Questionnaire; MEQ: Mystical Experience Questionnaire.

To evaluate the discriminant validity of the HQ, we investigated the correlation between the HS and MEQ scores. Consistent with our hypothesis, the HS demonstrated a moderate correlation with the MEQ (Figure 5). To further support discriminant validity, we assessed the correlation between the HS and the CEQ, which yielded no significant correlation (Figure 5). Correlations for all subscales can be found in the Supplemental Material (Table S5).

### Predictive and incremental validity

The predictive validity of the HQ was assessed by examining its ability to predict positive changes following the psychedelic experience, as measured by the PEQ. The HS significantly predicted positive long-term changes, measured by a composite score summarizing two subscales of the PEQ: “Positive Mood Changes” and “Positive Attitudes about Life or Self.” The regression analysis showed a significant effect (β = 1.02, *F*(1,466) = 224.6, *p* < 0.001), with the model explaining 33% of the variance (*R*^2^ = 0.33).

To evaluate the unique contribution of the HQ, we assessed its incremental validity in predicting positive life changes as measured by the PEQ. Two stepwise linear regressions were conducted to determine whether the HS added unique explanatory power beyond the contributions of the MEQ, and PIQ. In the initial model, positive PEQ scores were predicted using the MEQ and PIQ scores. This model was significant (*F*(2,465) = 361.1, *p* < 0.001), accounting for approximately 60.7% of the variance in positive PEQ scores (adjusted *R*^2^=0.61). The final model included HS as an additional predictor. This model was also significant (*F*(3,464) = 247.5, *p* < 0.001), and explained approximately 61.3% of the variance in positive PEQ scores (adjusted *R*^2^ = 0.61). The addition of HS led to a statistically significant but very small increase in explained variance (*ΔR*^2^ = 0.006, *F*(1,464) = 8.56, *p* = 0.004).

Next, a robust linear regression was conducted to evaluate the relationship between avoidant distress and negative changes in mood and attitudes about life or self measured with the PEQ. The model was statistically significant, with avoidant distress as significant predictor (*b* = 0.51, *t*(466) = 9.40, *p* < 0.001) which explained approximately 7.5% of the variance (pseudo-*R*^2^ = 0.075). To examine the incremental validity of the avoidant distress subscale, we used robust linear regression models predicting negative changes with CEQ scores. The model containing only the CEQ was significant (*b* = 9.92, *t*(466) = 10.26, *p* < 0.001) and explained 6.38% of the variance (pseudo-*R*^2^= 0.06). The model including both the CEQ and avoidant distress explained 13.57% of the variance (pseudo-*R*^2^= 0.14). The addition of the avoidant distress resulted in a significant improvement in model fit, Δ*R*^2^=7.18%, as confirmed by the likelihood ratio test (χ*2*(1) = 37.37, *p* < 0.001).

### Effect of set and setting on the helioscope effect

**Figure 6.**
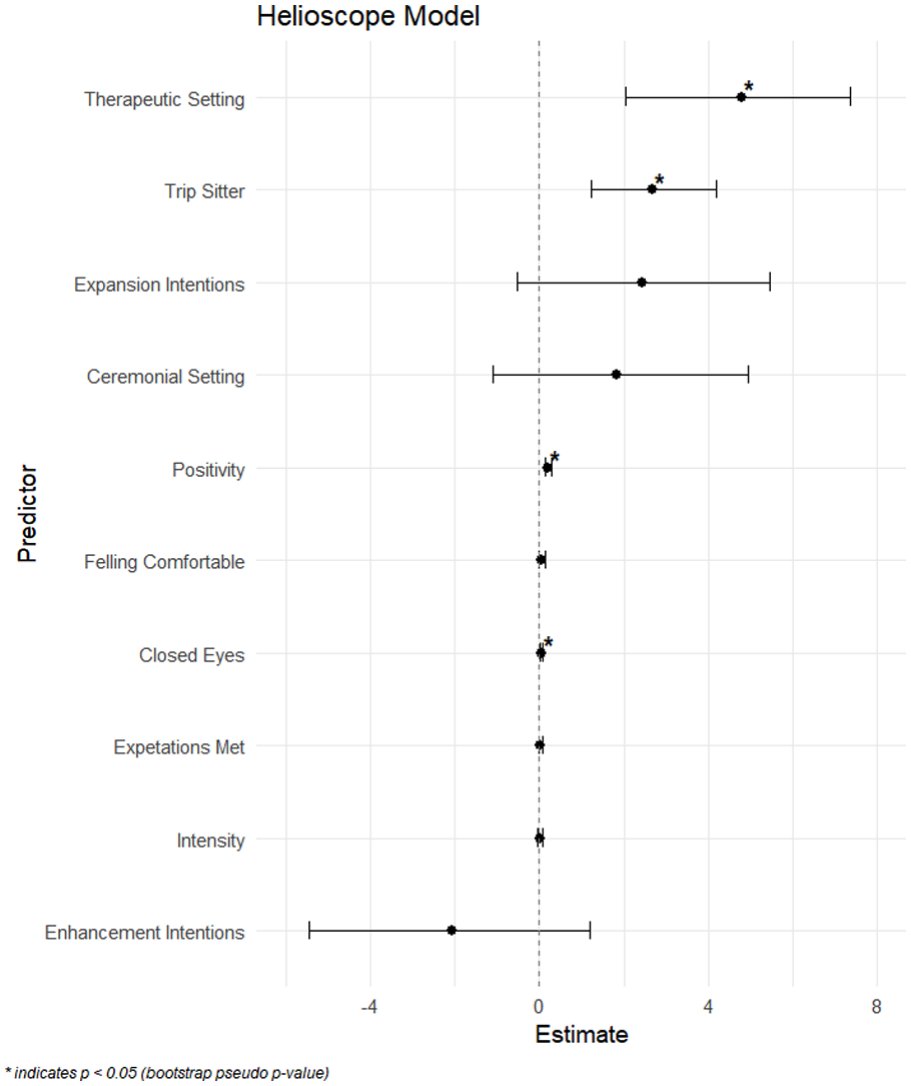
Coefficient plot showing the estimated effects of psychological and contextual predictors on helioscope score. Points indicate regression coefficients from a robust linear model; horizontal lines represent 95% bootstrap confidence intervals. Asterisks denote coefficients with bootstrap pseudo *p*-values <0.05.

To examine the influence of context variables on the helioscope effect and ADF, robust linear regression models with bootstrapping (1000 resamples) were fitted following variable selection using LASSO. Pseudo *p*-values and bias-corrected accelerated (BCa) confidence intervals were computed for inference. The final bootstrapped model identified several context variables significantly associated with the helioscope effect (see Figure 6). Most importantly, participants who reported having a trip sitter present exhibited significantly greater HS, *B* = 2.67, 95% BCa confidence interval (CI) [1.23, 4.20], *p* = 0.002. Positivity of the experience was also positively associated, *B* = 0.21, 95%CI [0.14, 0.29], *p* < 0.001. The percentage of closed eyes during the experience was a positive predictor, *B* = 0.04, 95%CI [0.01, 0.08], *p* = 0.016. Participants in therapeutic settings also showed elevated HS, *B* = 4.80, 95%CI [2.06, 7.36], *p* < 0.001. Other predictors identified by LASSO, including feelings of comfort, expectation matching, and enhancement or expansion intentions, did not reach statistical significance. The ten-predictor model explained 22% of the variance in HS (OLS benchmark: *R^2^* = 0.22, adj. *R^2^* = 0.20, *F*(10, 457) = 12.71, *p* < 0.001); the robust version showed a median absolute error of 10.9 scale points.

**Figure 7.**
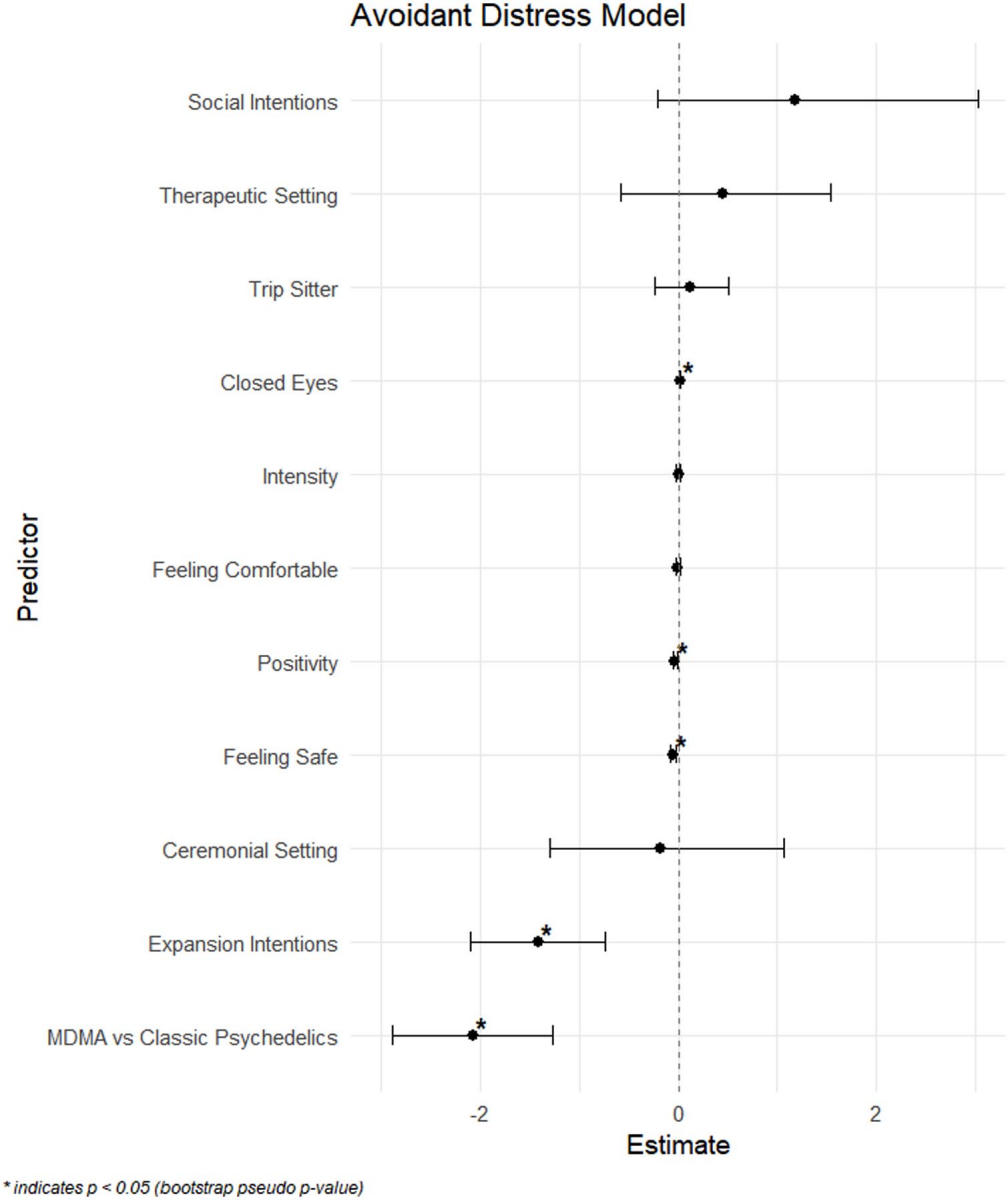
Coefficient plot depicting the relationship between selected predictors and avoidant distress factor. Estimates are derived from a robust linear regression with bootstrap resampling. Horizontal bars show 95% confidence intervals; asterisks denote coefficients with bootstrap pseudo *p*-values <0.05.

In the robust model predicting avoidant distress, several predictors emerged as statistically significant (see Figure 7). Most importantly, the use of MDMA (compared to classic psychedelics) was linked to significantly lower distress scores, *B* = −2.07, 95%CI [–2.89, –1.27], *p* < 0.001. Expansion-related intentions were negatively associated with distress, *B* = −1.42, 95%CI [–2.10, –0.74], *p* < 0.001. Higher self-reported feelings of safety also predicted reduced distress, *B* = −0.04, 95%CI [–0.07, –0.02], *p* < 0.001. Finally, keeping eyes closed was associated with slightly higher distress levels, *B* = 0.01, 95%CI [0.01, 0.03], *p* = 0.006. Notably, positivity of the experience was inversely associated with avoidant distress, *B* = −0.03, 95%CI [–0.05, –0.01], *p* = 0.004. Other context variables, including setting type and sitter presence, were not significantly related to distress outcomes. The corresponding model for avoidant distress accounted for 19% of the variance (OLS benchmark: *R^2^* = 0.19, *adj. R^2^* = 0.17, *F*(9, 460) = 11.8, *p* < 0.001); the robust fit yielded a median absolute error of 7 scale points.

## Discussion

### Consistency and validity of the scale

This study outlines the development and initial psychometric evaluation of the Helioscope Questionnaire, a novel scale designed to assess the perceived changes in detail and feelings of safety in the processing of difficult and/or traumatic experiences during psychedelic trips. It was developed to create a tool with scientific and clinical utility regarding the treatment of PTSD and various other mental disorders. The resulting self-report instrument consists of 21 items, which participants reported to be relevant to the psychedelic experience. The HQ demonstrated strong internal consistency, with three subscales assessing perceived safety (protection effect), engagement with difficult content (exposure effect), and distress-related avoidance (ADF). A total helioscope score was composed by adding the protection and exposure scores together to reflect the entirety of the helioscope effect (Hasler, 2022a, 2022b). The moderate correlation between protection and exposure suggests that these experiences are often linked, and psychedelic experiences can involve both a sense of safety and engagement with previously avoided topics, while the negative correlation between ADF and these subscales supports the idea that avoidance and distress represent opposing processes.

Convergent validity was supported by strong correlations with the PIQ, reinforcing the theoretical link between the helioscope effect and insight (Matthews and Chu, 1997; Singewald et al., 2015). The particularly strong association between reported exposure to difficult experiences and PIQ suggests that engagement with previously avoided experiences may facilitate insight, although the direction of causality remains unclear. As hypothesized, the ADF correlated moderately with the CEQ (Barrett et al., 2016), suggesting that while both constructs capture distress, the ADF specifically reflects avoidance and difficulty in processing traumatic memories, which may explain its additional predictive power for negative changes in mood and attitudes.

Discriminant validity was supported by a moderate correlation between the HQ total score and the MEQ (Barrett et al., 2015), indicating some overlap but also meaningful differences. A possible explanation for the overlap is that psychedelic experiences may involve distinct phases, allowing both processes to occur separately. Additionally, self-transcendence during mystical experiences may promote a sense of safety by creating psychological distance from distressing memories, making them more accessible to conscious awareness and open to therapeutic processing. Further supporting discriminant validity, the total HQ score showed no significant correlation with the CEQ, indicating no meaningful relationship.

Criterion validity and the potential clinical utility of the HQ and its subscales were indicated by their predictive power for both positive and negative changes in mood and attitudes. The helioscope score significantly predicted lasting positive changes, as measured by the PEQ (Griffiths et al., 2006) and further provided small but significant explanatory power for positive changes beyond constructs that previously established therapeutic utility, namely mystical experiences and psychological insight (Davis et al., 2021; Garcia-Romeu et al., 2019; Ko et al., 2022). This underscores the HQ’s potential as a tool for assessing therapeutic processes with lasting positive consequences in PAT and potentially other exposure-based therapies. Additionally, the ADF was a significant predictor of persistent negative changes, beyond what was explained by challenging aspects of the experience alone. This suggests that increasing feelings of distress and active avoidance of difficult memory processing during psychedelic experiences can contribute to lasting adverse effects that cannot be fully captured by the CEQ.

### The importance of context

These findings should be considered alongside the context in which the experience has taken place (Carhart-Harris et al., 2018). Importantly, MDMA use was associated with significantly lower avoidance and distress but did not predict higher helioscope scores relative to classic psychedelics. This pattern suggests that while MDMA may offer greater protection from negative emotional reactions, it may not facilitate deeper engagement with challenging psychological content. This finding aligns with prior research highlighting MDMA’s increasing use in trauma-focused interventions, where emotional safety and containment are critical therapeutic components (Smith et al., 2022; Yao et al., 2024). Furthermore, the presence of a trip sitter significantly predicted higher helioscope scores, reinforcing the importance of supported environments in facilitating therapeutic processes during PAT (Johnson et al., 2008). This was further substantiated by the finding that therapeutic settings, relative to recreational ones, were associated with greater helioscope scores and that feeling safe contributed significantly to reducing avoidant distress. Interestingly, while participants’ intention to expand their perspective was not significantly associated with higher helioscope scores, it was linked to reduced avoidance and distress. This pattern aligns with prior research suggesting that introspective motivations may buffer against challenging emotional reactions (Carbonaro et al., 2016; Haijen et al., 2018; Wolff et al., 2022). The finding that spending more time with eyes closed predicted stronger helioscope effects, yet also slightly amplified avoidant distress, suggests that introspective focus may be therapeutically valuable but warrants further investigation regarding potential downsides. Overall, this supports the notion that minimizing external visual input may facilitate deeper psychological processing and emotional integration during altered states, with caution to preparation and continuous care (Johnson et al., 2008). Finally, the general perceived positivity of the experience predicted a deeper engagement with difficult experiences and was also associated with less avoidance and distress, emerging as an important factor in psychedelic experiences.

### General therapeutic mechanisms and the helioscope effect

To effectively capture mental processes, reliable assessment tools are essential for advancing research and clinical practice. This study suggests that the HQ is a valid instrument for measuring altered memory processing during naturalistic psychedelic experiences, representing an additional step toward strengthening our understanding of therapeutic mechanisms in PAT. Comprehending these processes is crucial for developing interventions and evaluating why some treatments succeed while others do not. Previous psychedelic research has primarily focused on neurophysiological responses, molecular pathways, and acute subjective mechanisms underlying their therapeutic potential, with significant progress made in identifying key processes (van Elk and Yaden, 2022; Walther and van Schie, 2024).

Among these, the most-studied subjective effect is the mystical experience, which has been shown to predict positive therapeutic outcomes in both clinical and nonclinical populations (Ko et al., 2022). Yet, the concept’s operationalization is debated, especially concerning its cross-cultural application (Mosurinjohn et al., 2023; Yaden et al., 2024). Additional psychological mechanisms include awe (Hendricks, 2018) and ego-dissolution—a transient loss of self-boundaries (Nour et al., 2016)—which further contribute to the positive changes seen after psychedelic experiences, though distinguishing these constructs and the mystical-type experience empirically remains difficult (Taves, 2020). Given that psychological insight is a core element of most therapeutic approaches (Jennissen et al., 2018), it has also been proposed as a key process facilitated by psychedelics and linked to reductions in depression and alcohol consumption (Garcia-Romeu et al., 2019; Roseman et al., 2018b). Similarly, the Emotional Breakthrough Inventory (EBI) assesses states in which emotional barriers weaken, allowing cathartic release, which has been linked to increases in well-being (Roseman et al., 2019). In contrast to these positive psychological effects, Barrett et al. (2016) developed a scale assessing the challenging and distressing aspects of the psychedelic experience which have shown ambivalent long-term effects—either beneficial or detrimental depending on the intensity and duration of the challenging experience (Carbonaro et al., 2016).

These psychological mechanisms offer valuable insights into the therapeutic potential of psychedelics. However, research remains limited in bridging these effects with established psychotherapy frameworks (Yaden et al., 2024), particularly in understanding how psychedelics facilitate the re-emergence and processing of distressing memories—a mechanism central to trauma-focused therapies, including MDMA-AP (Mithoefer, 2015; Rauch et al., 2012). Here, the helioscope effect emerges as an impactful process in psychedelic experiences which have a strong foundation in neurological and psychotherapeutic research. This effect describes how psychedelics act as a protective filter, softening distressing memories for safer emotional integration (Hasler, 2022b) and can help to understand why psychedelics can be a supportive tool in diverse psychotherapeutic approaches (Wolff et al., 2024; Yao et al., 2024).

The helioscope effect provides a valuable framework for understanding how psychedelic treatments support self-exploration, emotional processing, and lasting therapeutic change, including core processes in PAT that overlap with *problem actualization*—the activation and conscious engagement with difficult personal content proposed by Grawe as a basic psychotherapeutic process (Davis et al., 2021; Grawe, 1997; Hasler, 2022b; Knudsen, 2023; Roseman et al., 2019; Weiss et al., 2023). It is also especially relevant in trauma therapy, where overwhelming memories often require prior *resource activation* to avoid symptom exacerbation (Grawe, 1997; Larsen et al., 2010). Psychedelics can enable this by creating a safe emotional space in which challenging experiences can emerge without overwhelming the individual, thus facilitating deep emotional processing. They also promote cognitive flexibility (Davis et al., 2020; Doss et al., 2021), positive mood (Gandy, 2019), and self-distancing (Nour et al., 2016), which can help patients access and work through traumatic material. These mechanisms enhance the therapeutic relationship and support *mastery*, other processes proposed by Grawe, as patients begin to actively face and transform situations that were previously too difficult (Bouso et al., 2008; Dolder et al., 2016; Grawe, 1997; Oehen and Gasser, 2022; White, 2014). Overall, the helioscope effect aligns closely with Grawe’s model and offers a structured lens for understanding therapeutic change in psychedelic-assisted settings. While Grawe’s concept are very general and broad, the helioscope concept provides a more specific framework to study trauma processing in psychedelic and other kinds of psychotherapies.

### Fear memory reconsolidation: Biological foundation of the HQ

In contrast to the concepts of mystical experiences, insight, and emotional breakthrough, the helioscope concept has the advantage that it is based on basic psychophysiology and has the potential to bridge the gap between clinical and preclinical science. A potential mechanism behind reductions of stress and arousal during the processing of aversive content is the acute reduction of the activity and responsiveness of the amygdala to fearful stimuli (Bedi et al., 2009; Gamma et al., 2000; Kraehenmann et al., 2015; Mueller et al., 2017). Recent work suggests that this is associated with greater reduction in psychiatric symptoms (Mertens et al., 2020; Roseman et al., 2018a). Additionally, increased oxytocin in response to MDMA may foster empathy and increase openness, supporting the therapeutic process (Sottile and Vida, 2022; Vizeli and Liechti, 2018; Wagner et al., 2017).

Highlighting the translational applicability of the HQ, preclinical studies have demonstrated that MDMA and classic psychedelics promote fear extinction and memory reconsolidation, potentially enabling the attenuation of traumatic memories into less distressing perceptions (Cameron et al., 2018, 2019; Catlow et al., 2013; Curry et al., 2018; Du et al., 2023; Kelly et al., 2024; Werle et al., 2024; Young et al., 2015, 2017) and initial clinical research suggests a translation of these findings into humans (Vizeli et al., 2022). A recent review further suggested that psychedelics may acutely enhance learning of semantic memories that could facilitate the integration of trauma memories and disrupt maladaptive beliefs (Doss et al., 2024). This builds a sound foundation in neurobiological and preclinical research, which is lacking for other scales used in psychedelic research. These findings emphasize the HQ’s potential to bridge gaps in current assessment tools and enhance the understanding of therapeutic processes in PAT.

### Complementary effects—Avoidance and distress

Despite overall promising results, not all individuals undergoing psychedelic experiences derive lasting positive effects, and for some, these experiences can be among the most challenging of their lives (Carbonaro et al., 2016). A representative survey found that 2.6% of participants sought professional treatment following a distressing psychedelic experience (Simonsson et al., 2023). More specifically, not all psychedelic sessions involving the re-experiencing of traumatic memories lead to successful emotional processing or symptom reduction (Breeksema et al., 2022) and some might even lead to persistent harm (Evans et al., 2023).

Noteworthy, our findings suggest that distress and active avoidance during the reemergence of difficult content may facilitate these negative effects. This may explain why in MDMA trials for PTSD several cases of prolonged adverse effects were reported, which were attributed to emerging traumatic memories that could not be adequately processed during treatment (Lindsay, 2022; McNamee et al., 2023). Similarly, long-term adverse events have been documented in psilocybin trials, particularly when re-experienced traumatic memories could not be positively integrated (Anderson et al., 2020; Watts et al., 2017). These cases underscore the importance of systematically assessing adverse effects, both to enhance safety and to facilitate the integration of psychedelics into medical practice. The HQ stands out in this regard, as it not only evaluates beneficial aspects but also includes adverse effects, a factor often overlooked in other assessment tools. This aligns with the approach of the Swiss Psychedelic Side Effects Inventory (Calder and Hasler, 2023), which was specifically developed to monitor long-term adverse reactions, highlighting the importance of tracking both positive and negative responses in PAT.

Our findings show that the ADF significantly predicts negative long-term changes, suggesting that a perceived lack of safety in processing challenging emotions—or an active avoidance of distressing content—may contribute to lasting adverse effects in both clinical trials and naturalistic settings. This aligns with psychotherapeutic concepts which see avoidance as detrimental to the therapeutic process (Grawe, 1997; Wolff et al., 2020). Future clinical studies should assess emotional avoidance, as it may not only reduce the therapeutic benefits but also increase the risk of persistent negative effects, underscoring the need to develop strategies that facilitate safe emotional engagement and integration. Recognizing and addressing these negative influences is essential for refining PAT and improving harm reduction strategies (Carbonaro et al., 2016; Evans et al., 2023; Simonsson et al., 2023).

### Future research

While the HQ addresses a gap in assessing altered memory processing, continued investigation is needed to fully characterize this state. The EBI (Roseman et al., 2019), the PIQ (Davis et al., 2021), and the HQ seem to assess phenomena that are all related to an improvement in mood and a possible reevaluation of the personal and social identity. Considering some overlap between mystical experience and the helioscope effect, assessing the loss of ego-boundaries and self-transcendence in particular (Nour et al., 2016) and its relationship to the HQ could bear also further insight into how these mechanisms interact. To assess all these measures in one sample and evaluate the relationship they have with one another can offer valuable insight.

To ascertain the HQ’s relevance in therapeutic contexts, it is essential to evaluate its effectiveness through clinical trials involving various patient groups. Researchers can leverage this tool to deepen their understanding of why psychedelics may serve as an effective adjunct to conventional psychotherapy. To further examine the role of the helioscope effect in psychotherapeutic process, studies should simultaneously assess the General Mechanisms of Change Questionnaire (Wolff et al., 2024). This way a deeper understanding of general therapeutic mechanisms in PAT can be gathered. Moreover, the HQ presents an opportunity to explore the existence of the helioscope effect in alternative trauma-focused treatments, such as eye movement desensitization and reprocessing and mindfulness-based treatments for PTSD, thereby helping to determine whether this process is substance-specific or a broader therapeutic mechanism. Finally, the helioscope concept may serve as a translational framework: by quantifying comparable psychological states, it can tentatively align behavioral and pharmacological readouts in animal models with clinical observations in humans, thereby helping to elucidate the neurobiological mechanisms underlying psychedelic therapy.

### Limitations

This study is subject to several methodological limitations. First, the data were collected via a retrospective, self-reported, and anonymous online survey, introducing potential biases and variability. Factors such as internet access, recall bias, individual mood at the time of survey completion, and self-selection tendencies may have influenced responses.

Second, recruitment was targeted at individuals with prior psychedelic use, likely resulting in selection bias. Participants engaged in psychedelic-related communities may overestimate the positivity of the experiences and the connection between altered experience processing and persistent positive changes. The advertisement specified that memory processing was an area of interest, adding to the selection bias. Additionally, most respondents were U.S. citizens, which may reflect differences in social media use and interest in psychedelic research, further limiting generalizability. Finally, a high dropout rate (48.2%) during survey completion could introduce selection bias.

Third, while this study examined positive changes following psychedelic use, the Persisting Effects Questionnaire and its subscales lack formal validation (Griffiths et al., 2006). Therefore, it remains unclear whether subjective reports of positive changes translate into real-life improvements.

Fourth, our sample primarily consisted of young Caucasian males, consistent with previous studies using similar recruitment strategies (Barrett et al., 2016; Davis et al., 2021). Consequently, caution should be exercised when generalizing these findings to broader demographic groups.

Fifth, a subset of responses was coded “not applicable/cannot say” which we treated as missing and replaced by multiple imputation. Although this procedure is statistically appropriate and sensitivity checks showed the factor structure was unchanged, we cannot rule out residual bias: some “not applicable” entries may reflect a true absence of experience and imputing them could slightly inflate average HQ scores. Some “not applicable/cannot say” entries may reflect missing not at random (MNAR). While our diagnostics support missing at random as a working assumption, we acknowledge potential MNAR. Replication with designs that minimize such skips, or that analyze them as a distinct response category, will help to clarify any impact.

Finally, this study did not assess participants’ mental health history, limiting the applicability of findings to clinical populations. Additionally, individuals with PTSD or past trauma were not specifically targeted, restricting conclusions regarding psychedelics’ effects on trauma processing.

## Conclusion

This study introduces the Helioscope Questionnaire, a valid and reliable tool for assessing altered memory processing during psychedelic experiences, highlighting important therapeutic mechanisms in PAT. The HQ effectively measures perceived safety, engagement with difficult memories, and avoidant distress, closely aligning with therapeutic mechanisms in psychological treatments. Its predictive validity for both positive and negative changes in mood and attitudes underscores the critical role of addressing difficult memories and emotional avoidance in therapeutic contexts. The concept of the helioscope effect integrates insights from neurobiological and psychological research, providing a structured approach to evaluate therapeutic efficacy. Future research should further examine the HQ’s clinical utility, explore interactions with other psychological mechanisms, and investigate its broader applicability across diverse therapeutic modalities.

## Supplemental Material

sj-docx-1-jop-10.1177_02698811251397306 – Supplemental material for The helioscope effect: A new framework for evaluating trauma-related memory processing in psychedelic experiences Supplemental material, sj-docx-1-jop-10.1177_02698811251397306 for The helioscope effect: A new framework for evaluating trauma-related memory processing in psychedelic experiences by Vincent J. Diehl, Abigail E. Calder and Gregor Hasler in Journal of Psychopharmacology
