# Supplementary material for: The helioscope effect: A new framework for evaluating trauma-related memory processing in psychedelic experiences
Source: J Psychopharmacol. 2025 Dec 31;40(5):769–84. doi: 10.1177/02698811251397306 (PMC13310273; doi:10.1177/02698811251397306)
Supplement: sj-docx-1-jop-10.1177_02698811251397306 – Supplemental material for The helioscope effect: A new framework for evaluating trauma-related memory processing in psychedelic experiences [file sj-docx-1-jop-10.1177_02698811251397306.docx]

# Supplementary Material

## Methods

### Data Analysis

We report that Missing Completely at Random (MCAR) was not supported (Little’s test; MissMech with ≥2-case patterns) and that we proceeded under a Missing At Random (MAR) assumption with MICE using all items. Item-wise logistic models were calculated, which showed that missingness in several items was related to other observed HQ responses. However, this is consistent with MAR when those variables are included as predictors in the imputation model.

To test the robustness of our approach, we evaluated measurement equivalence in two steps, beginning with missing‑data handling. Firstly, we checked the impact of the imputation: we ran the full EFA workflow on the complete‑case sample and on the first imputed dataset. We compared solutions via Tucker’s factor‑congruence (after optimal factor matching) and by inspecting |Δh²| ≥ .10 for communalities; eigenvalues and cumulative variance were also examined. Additionally, we compared the distribution of the imputed and original datasets as well as the mean value of each variable. Secondly, we checked for differences between languages by repeating the same procedure was for the English and German subsamples. All analyses were carried out in R using psych, lavaan, semTools, and tidyverse.

## Results

### Exploratory Factor Analysis

To begin, we imputed missing values in the HQ and compared the imputed with the original dataset. We evaluated whether item nonresponse was MCAR using the *MissMech* test based on missing-data patterns. With a setting that retains rare patterns with at least two cases (del.lesscases = 1), 15 distinct patterns comprising N = 323 observations were analyzed. The non-parametric homoscedasticity test rejected MCAR (*p* = 0.049). Thus, MCAR was not supported; we proceeded under a MAR working assumption for handling missing data. Mean values as well as the distribution of the data in the questionnaire remained similar (Table S3). We then compared the imputed data with complete cases on their factor structure. Tucker’s congruence coefficients between the two loading patterns were .99, .96, and .98 for Factors 1–3, respectively, indicating virtually identical factor solutions (φ > .95; (Lorenzo-Seva and Ten Berge, 2006). Item communalities changed by no more than |.09| (MΔ = .03), confirming that the imputation procedure did not materially alter the factor structure. We thus used the imputed data for analysis. Next, we conducted Mann-Whitney U tests which revealed no significant differences between the German and English datasets on most items after Benjamini-Hochberg correction (p > 0.05; Table S4). Only two items differed, one of which was later excluded from factor analysis. Additionally, we compared factor structure between the languages. Both groups were analyzed with a three-factor model. Tucker’s factor-congruence coefficients indicated that Factor 1 replicated very well across languages (φ = .94), whereas Factors 2 and 3 showed fair similarity (φ = .71 and .64, respectively). Collectively, the results support configural equivalence for the dominant factor but point to small language-specific nuances in the remaining dimensions. As the remaining difference was minimal and the overall response structure comparable, we combined both datasets to enhance statistical power and analytical robustness.

## Tables

Table S1 Original pool of items in the Helioscope Questionnaire prior to factor analysis

|  |  | Mean (SD) |
| --- | --- | --- |
| Item 1 | The session allowed me to avoid difficult memories and topics less. | 3.17 (1.40) |
| Item 2 | During the session, I could endure traumatic or difficult memories, feelings, or thoughts better than usual. | 3.89 (1.31) |
| Item 3 | The session helped me to let go of difficult experiences or memories, or to accept them better. | 4.01 (1.11) |
| Item 4 | During the session, experienced a sense of security and safety which allowed me to engage with stressful topics. | 3.84 (1.21) |
| Item 5 | During the session, I avoided difficult feelings or memories more than usual. | 2.02 (1.18) |
| Item 6 | Memories, sensations, or thoughts which normally cause unpleasant feelings were easier to endure during the session. | 3.79 (1.45) |
| Item 7 | The session helped me address crucial life questions that I otherwise avoid. | 3.84 (1.12) |
| Item 8 | The session gave me access to memories or feelings that were not fully conscious before. | 3.85 (1.28) |
| Item 9 | During the session, I was surrounded by a protective armor that kept memories and feelings from hurting me. | 2.80 (1.34) |
| Item 10 | During the session, I saw difficult situations and experiences from my past more clearly and with more detail. | 3.48 (1.26) |
| Item 11 | During the session, difficult memories or feelings burdened me in an unpleasant way. | 2.19 (1.29) |
| Item 12 | The session allowed me to engage with distressing memories, emotions, or thoughts that I normally cannot bear. | 3.46 (1.25) |
| Item 13 | During the session I could bear difficult physical sensations better than usual. | 3.27 (1.24) |
| Item 14 | Because the session allowed me to confront the challenging and distressing aspects of my life, I was able to process them better. | 4.05 (1.05) |
| Item 15 | During the session, I experienced stress that made it more difficult for me to engage with psychologically important topics. | 2.01 (1.25) |
| Item 16 | The session helped me deal with sickness and death without being overwhelmed by them. | 3.46 (1.28) |
| Item 17 | After the session, I was better able to withstand distressing memories, thoughts, or feelings. | 3.94 (1.03) |
| Item 18 | During the session I was less able to withstand difficult emotions or memories than usual. | 2.10 (1.25) |
| Item 19 | During the session I felt that I possessed a protective filter which allowed me to withstand difficult emotions or memories. | 3.00 (1.33) |
| Item 20 | During the session I was able to confront negative memories without being overwhelmed by them. | 3.64 (1.18) |
| Item 21 | During the session I felt like I was being guided to those traumatic experiences which were ready to be psychologically processed. | 3.39 (1.30) |
| Item 22 | The session helped me confront existential issues without being overwhelmed by them. | 3.86 (1.20) |
| Item 23 | The session helped me to better understand my history of trauma. | 3.70 (1.22) |
| Item 24 | During the session, I avoided difficult issues and experiences even more than usual. | 1.85 (1.45) |
| Item 25 | The relaxation I felt during the session helped me deal with difficult life events. | 3.75 (1.17) |

Table S2 Intentions for psychedelic experience.

| Group | Intention | Mean HS | SD HS |
| --- | --- | --- | --- |
| Social | It is part of my social, religious, or cultural identity | 62.55 | 11.70 |
|  | To connect with other people or enhance socializing |  |  |
| Conformity | To fit in with a group | 61.80 | 11.59 |
| Coping | To forget my worries or relieve negative emotions | 62.33 | 5.79 |
| Expansion | Introspection; to help me learn about myself | 64.15 | 12.25 |
|  | To understand things differently |  |  |
|  | To help me work through my problems |  |  |
|  | To have a spiritual experience |  |  |
| Enhancement | To party or get "messed up" | 57.39 | 14.00 |
|  | Out of curiosity |  |  |
|  | I was bored |  |  |
|  | For relaxation |  |  |
|  | To enjoy the sensations |  |  |
|  | To enhance my creativity |  |  |

Note: HS = Helioscope Score

Table S3 Imputed vs Original datasets. Compared for distribution and mean item value.

| Variable | Mean (Obs.) | SD (Obs.) | Mean (Imp.) | SD (Imp.) | KS p-value | T p-value |
| --- | --- | --- | --- | --- | --- | --- |
| Item 1 | 3.16 | 1.40 | 3.32 | 1.27 | 0.975 | 0.476 |
| Item 2 | 3.89 | 1.14 | 3.86 | 1.06 | 1 | 0.855 |
| Item 3 | 4.00 | 1.10 | 4.10 | 1.16 | 0.996 | 0.616 |
| Item 4 | 3.85 | 1.21 | 3.62 | 1.30 | 0.98 | 0.375 |
| Item 5 | 2.02 | 1.18 | 1.95 | 1.08 | 0.951 | 0.764 |
| Item 6 | 3.80 | 1.14 | 3.59 | 1.21 | 0.926 | 0.352 |
| Item 7 | 3.83 | 1.12 | 3.91 | 1.15 | 0.955 | 0.719 |
| Item 8 | 3.84 | 1.28 | 4.04 | 1.37 | 0.904 | 0.476 |
| Item 9 | 2.80 | 1.33 | 2.90 | 1.47 | 0.996 | 0.726 |
| Item 10 | 3.48 | 1.27 | 3.50 | 1.17 | 0.99 | 0.916 |
| Item 11 | 2.19 | 1.29 | 2.25 | 1.53 | 0.745 | 0.884 |
| Item 12 | 3.45 | 1.24 | 3.51 | 1.37 | 0.937 | 0.776 |
| Item 13 | 3.28 | 1.25 | 3.22 | 1.22 | 1 | 0.743 |
| Item 14 | 4.08 | 1.05 | 3.76 | 1.08 | 0.105 | 0.076 |
| Item 15 | 2.01 | 1.24 | 2.22 | 1.35 | 0.767 | 0.514 |
| Item 16 | 3.40 | 1.31 | 3.63 | 1.15 | 0.427 | 0.077 |
| Item 17 | 3.94 | 1.04 | 4.00 | 0.84 | 0.895 | 0.752 |
| Item 18 | 2.10 | 1.25 | 2.05 | 1.23 | 0.728 | 0.866 |
| Item 19 | 3.00 | 1.33 | 2.94 | 1.35 | 1 | 0.803 |
| Item 20 | 3.65 | 1.18 | 3.50 | 1.18 | 0.95 | 0.453 |
| Item 21 | 3.40 | 1.31 | 3.30 | 1.25 | 0.919 | 0.594 |
| Item 22 | 3.86 | 1.20 | 3.91 | 1.29 | 0.994 | 0.804 |
| Item 23 | 3.69 | 1.22 | 3.75 | 1.18 | 0.558 | 0.746 |
| Item 24 | 1.85 | 1.15 | 2.00 | 1.18 | 0.896 | 0.684 |
| Item 25 | 3.74 | 1.17 | 3.90 | 1.17 | 0.785 | 0.563 |

*Note: KS p-value = Kolmogorov-Smirnoff Test; T p-value = t-test; Obs. = observed; Imp. = imputed.*

Table S4 Comparison of German vs English dataset. p-value for Wilcoxon rank sum test adjusted with Benjamin Hochberg correction.

| Variable | Mean (Eng.) | SD (Eng.) | Mean (Ger.) | SD (Ger.) | U-Statistic | p-Value | Adj. p-Value |
| --- | --- | --- | --- | --- | --- | --- | --- |
| Item 1 | 3.05 | 1.386 | 3.619 | 1.396 | 11244.5 | 0.001 | 0.014 |
| Item 2 | 3.90 | 1.102 | 3.872 | 1.303 | 13072 | 0.622 | 0.822 |
| Item 3 | 4.01 | 1.079 | 3.987 | 1.204 | 13441.5 | 0.745 | 0.887 |
| Item 4 | 3.83 | 1.223 | 3.951 | 1.121 | 14074 | 0.491 | 0.822 |
| Item 5 | 2.10 | 1.21 | 1.72 | 1.01 | 18592 | 0.005 | 0.045 |
| Item 6 | 3.82 | 1.11 | 3.73 | 1.27 | 15038.5 | 0.798 | 0.906 |
| Item 7 | 3.82 | 1.16 | 3.89 | 0.93 | 14871 | 0.963 | 0.994 |
| Item 8 | 3.80 | 1.29 | 4.00 | 1.20 | 14295 | 0.275 | 0.822 |
| Item 9 | 2.78 | 1.33 | 2.89 | 1.35 | 13963.5 | 0.507 | 0.822 |
| Item 10 | 3.51 | 1.24 | 3.36 | 1.39 | 14018.5 | 0.469 | 0.822 |
| Item 11 | 2.20 | 1.30 | 2.17 | 1.24 | 15868.5 | 0.994 | 0.994 |
| Item 12 | 3.48 | 1.24 | 3.33 | 1.23 | 14808 | 0.295 | 0.822 |
| Item 13 | 3.30 | 1.24 | 3.16 | 1.27 | 12142.5 | 0.42 | 0.822 |
| Item 14 | 4.11 | 1.02 | 3.95 | 1.14 | 14795 | 0.305 | 0.822 |
| Item 15 | 2.06 | 1.27 | 1.79 | 1.09 | 17282 | 0.079 | 0.396 |
| Item 16 | 3.40 | 1.28 | 3.43 | 1.45 | 9505 | 0.604 | 0.822 |
| Item 17 | 3.94 | 1.05 | 3.95 | 0.99 | 15293 | 0.963 | 0.994 |
| Item 18 | 2.19 | 1.29 | 1.70 | 1.00 | 18423.5 | 0.001 | 0.014 |
| Item 19 | 2.96 | 1.31 | 3.22 | 1.43 | 12373.5 | 0.102 | 0.425 |
| Item 20 | 3.66 | 1.19 | 3.61 | 1.17 | 14608.5 | 0.625 | 0.822 |
| Item 21 | 3.38 | 1.31 | 3.50 | 1.33 | 11618 | 0.462 | 0.822 |
| Item 22 | 3.86 | 1.22 | 3.82 | 1.13 | 14525.5 | 0.503 | 0.822 |
| Item 23 | 3.72 | 1.19 | 3.57 | 1.38 | 12588 | 0.604 | 0.822 |
| Item 24 | 1.91 | 1.19 | 1.57 | 0.88 | 18103 | 0.022 | 0.138 |
| Item 25 | 3.76 | 1.16 | 3.68 | 1.22 | 15447 | 0.665 | 0.831 |
|  |  |  |  |  |  |  |  |

Note: Ger. = German ; Eng. = English; Adj. = Adjusted

Table S5 Spearman correlation of HQ subscales with MEQ, PIQ and CEQ. P-values adjusted with Benjamin Hochberg correction for multiple comparisons.

| HQ Subscale | Scale | Spearman  Correlation | | p-value | Adj.  p-value |
| --- | --- | --- | --- | --- | --- |
| Avoidant Distress Factor | MEQ Total Score | -0.04 | 0.390 | | 0.390 |
|  | PIQ Total Score | -0.09 | 0.056 | | 0.083 |
|  | CEQ Total Score | 0.30 | <0.001 | | <0.001 |
| Protection Effect | MEQ Total Score | 0.39 | <0.001 | | <0.001 |
|  | PIQ Total Score | 0.44 | <0.001 | | <0.001 |
|  | CEQ Total Score | -0.08 | 0.144 | | 0.125 |
| Exposure Effect | MEQ Total Score | 0.41 | <0.001 | | <0.001 |
|  | PIQ Total Score | 0.70 | <0.001 | | <0.001 |
|  | CEQ Total Score | 0.23 | <0.001 | | <0.001 |

Note: MEQ = Mystical Experience Questionnaire; PIQ = Psychological Insight Questionnaire; CEQ = Challenging Experience Questionnaire; Adj. = Adjusted

## Figures

Figure S1 Polychoric correlation matrix of HQ items. X indicates correlation < 0.3 or > -0.3.


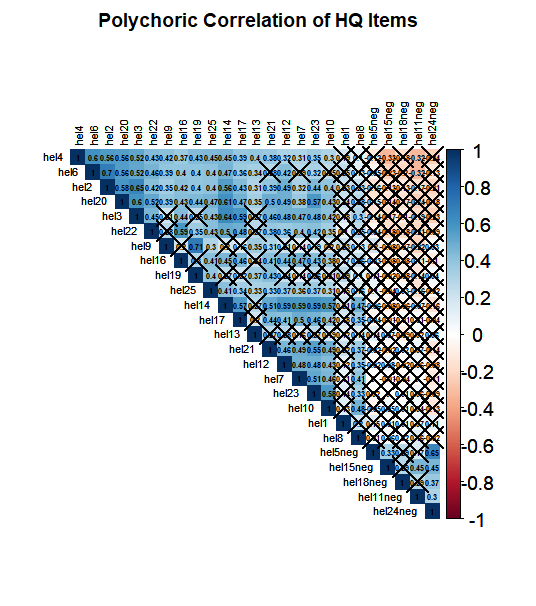


Figure S2 Final Helioscope Questionnaire and scoring manual in English

**Helioscope Questionnaire**

Please answer the following questions about your psychedelic session.

Please indicate how strongly you agree with each statement, from “completely disagree” on the left to “completely agree” on the right.

Some of the statements are about difficult experiences. By “difficult experience,” we mean memories or emotions that are stressful or burdensome, and which cause fear, pain or uncomfortable feelings.

Completely disagree

Rather disagree

Neither agree nor disagree/neutral

Rather agree

Completely agree

| Item |  |
| --- | --- |
| 1. *During the session, I could endure traumatic or difficult memories, feelings, or thoughts better than usual.* |  |
| 1. *The session helped me to let go of difficult experiences or memories, or to accept them better.* |  |
| 1. *During the session, I experienced a sense of security and safety which allowed me to engage with stressful topics.* |  |
| 1. *During the session, I avoided difficult feelings or memories more than usual.* |  |
| 1. *Memories, sensations, or thoughts which normally cause unpleasant feelings were easier to endure during the session.* |  |
| 1. *The session helped me address crucial life questions that I otherwise avoid.* |  |
| 1. *The session gave me access to memories or feelings that were not fully conscious before.* |  |
| 1. *During the session, I was surrounded by a protective armor that kept memories and feelings from hurting me.* |  |
| 1. *During the session, I saw difficult situations and experiences from my past more clearly and with more detail.* |  |
| 1. *The session allowed me to engage with distressing memories, emotions, or thoughts that I normally cannot bear.* |  |
| 1. *During the session I could bear difficult physical sensations better than usual.* |  |
| 1. *Because the session allowed me to confront the challenging and distressing aspects of my life, I was able to process them better.* |  |
| 1. *During the session, I experienced stress that made it more difficult for me to engage with psychologically important topics.* |  |
| 1. *After the session, I was better able to withstand distressing memories, thoughts, or feelings.* |  |
| 1. *During the session I was less able to withstand difficult emotions or memories than usual.* |  |
| 1. *During the session I felt that I possessed a protective filter which allowed me to withstand difficult emotions or memories.* |  |
| 1. *During the session I was able to confront negative memories without being overwhelmed by them.* |  |
| 1. *During the session I felt like I was being guided to those traumatic experiences which were ready to be psychologically processed.* |  |
| 1. *The session helped me to better understand my history of trauma.* |  |
| 1. *During the session, I avoided difficult issues and experiences even more than usual.* |  |
| 1. *The relaxation I felt during the session helped me deal with difficult life events.* |  |

*©* Copyright *Gregor Hasler, Universität Freiburg, Schweiz; Lake Lucerne Institute, Schweiz*

**Scoring Manual for the Helioscope Questionnaire**

This scoring manual is designed to help you score a questionnaire with items scored from 1 to 5. Please follow these guidelines to calculate scores accurately.

- **1: Completely Disagree:** Score of 1 when the respondent strongly disagrees with the statement or question.
- **2: Rather Disagree:** Score of 2 when the respondent leans towards disagreeing but is not completely in disagreement.
- **3: Neither Agree Nor Disagree / Neutral:** Score of 3 when the respondent neither agrees nor disagrees with the statement. This represents a neutral response.
- **4: Rather Agree:** Score of 4 when the respondent leans towards agreeing but is not completely in agreement.
- **5: Completely Agree:** Score of 5 when the respondent strongly agrees with the statement or question.

**Subscale Scores**

The Helioscope Questionnaire has three subscales which can be summarized independently.

Helioscope Exposure Effect: Items 2, 6, 7, 9, 10, 12, 14, 18, 19

Helioscope Protection Effect: Items 1, 3, 5, 8, 11, 16, 17, 21

Avoidant Distress Factor: Items 4, 13, 15, 20 (Max. score 20).

**Total Score**

To yield a total helioscope score the values of the Protection Effect and the Exposure Effect are added together.

Maximum possible score is 85 and the lowest possible score is 17.

By following this coding manual, you will accurately score the questionnaire. If you have any further questions or need assistance, please feel free to reach out to

[gregor.hasler@unifr.ch](mailto:gregor.hasler@unifr.ch) or [vincent.diehl@unifr.ch.](mailto:vincent.diehl@unifr.ch)

Figure S3 Final Helioscope Questionnaire and scoring manual in German

**Helioskop-Fragebogen**

Wir bitten Sie, folgende Fragen zur letzten Sitzung zu beantworten.

Bitte kreuzen Sie an, wie stark Sie den Aussagen jeweils zustimmen, wobei links gar keine Zustimmung und rechts völlige Zustimmung bedeutet.

Einige der Aussagen beziehen sich auf schwierige oder traumatische Erfahrungen. Darunter verstehen wir stark belastende Ereignisse oder Situationen, welche Angst, Schmerz oder unangenehme Gefühle auslösen.

stimme überhaupt nicht zu

stimme eher nicht zu

teils / teils oder neutral

stimme eher zu

stimme völlig zu

| Item |  |
| --- | --- |
| 1. *Während der Sitzung konnte ich traumatische oder herausfordernde Erinnerungen, Gefühle oder Gedanken besser aushalten als sonst.* |  |
| 1. *Die Sitzung half mir, schwierige Erfahrungen oder Erinnerungen loszulassen oder sie besser zu akzeptieren.* |  |
| 1. *Während der Sitzung empfand ich ein Gefühl von Geborgenheit und Sicherheit, das es mir erlaubte, mich mit belastenden Themen auseinanderzusetzen.* |  |
| 1. *In der Sitzung erzeugten schwierige Gefühle oder Erinnerungen noch mehr Abwehr und Vermeidung als sonst.* |  |
| 1. *Erinnerungen, Empfindungen oder Gedanken, die normalerweise bei mir unangenehme Gefühle auslösen, waren während der Sitzung besser aushaltbar.* |  |
| 1. *Die Sitzung half mir, mich mit entscheidenden Lebensfragen zu beschäftigen, die ich sonst vermeide.* |  |
| 1. *Die Sitzung verschaffte mir Zugang zu Erinnerungen oder Gefühlen, die mir vorher nicht ganz bewusst waren.* |  |
| 1. *Während der Sitzung umgab mich ein Schutzmantel, der mich vor Verletzungen durch Erinnerungen und Gefühle bewahrte.* |  |
| 1. *In der Sitzung sah ich schwierige Situationen und Erlebnisse meiner Vergangenheit klarer und mit mehr Details.* |  |
| 1. *Die Sitzung ermöglichte mir die Beschäftigung mit belastenden Erinnerungen, Gefühlen oder Gedanken, die ich vorher nicht aushalten konnte.* |  |
| 1. *Während der Sitzung konnte ich schwierige körperliche Empfindungen besser aushalten als sonst.* |  |
| 1. *Da die Sitzung mir erlaubte, den schwierigen und belastenden Aspekten meines Lebens in die Augen zu sehen, konnte ich diese besser verarbeiten.* |  |
| 1. *Während der Sitzung erlebte ich Stress, der mir die Auseinandersetzung mit psychologisch wichtigen Themen erschwerte.* |  |
| 1. *Nach der Sitzung gelang es mir besser, belastende Erinnerungen, Gedanken oder Gefühle auszuhalten.* |  |
| 1. *Während der Sitzung konnte ich schwierige Emotionen oder Erinnerungen schlechter aushalten als sonst.* |  |
| 1. *In der Sitzung hatte ich das Gefühl, einen Schutzfilter zu besitzen, der es mir erlaubte, schwierige Emotionen oder Erinnerungen auszuhalten.* |  |
| 1. *In der Sitzung gelang es mir, mich negativen Erinnerungen zu stellen, ohne von ihnen überwältigt zu werden.* |  |
| 1. *Ich hatte das Gefühl, dass ich in der Sitzung zu denjenigen traumatischen Erfahrungen geführt wurde, die reif für die psychische Verarbeitung waren.* |  |
| 1. *Die Sitzung half mir, meine traumatische Geschichte besser zu verstehen.* |  |
| 1. *In der Sitzung bin ich schwierigen Themen und Erfahrungen noch mehr aus dem Wege gegangen als sonst.* |  |
| 1. *Die Entspannung während der Sitzung half mir, mich mit schwierigen Lebensereignissen auseinanderzusetzen.* |  |

*©* Copyright *Gregor Hasler, Universität Freiburg, Schweiz; Lake Lucerne Institute, Schweiz*

**Bewertungshandbuch für den Helioskop-Fragebogen**

Dieses Bewertungshandbuch soll Ihnen dabei helfen, einen Fragebogen mit Items zu bewerten, die mit 1 bis 5 bewertet werden. Bitte befolgen Sie diese Richtlinien, um die Bewertungen genau zu berechnen.

- **1: Stimme überhaupt nicht zu:** Bewertung mit 1, wenn der Befragte der Aussage oder Frage entschieden widerspricht.
- **2: Stimme eher nicht zu**: Bewertung mit 2, wenn der Befragte eher nicht zustimmt, aber nicht völlig widerspricht.
- **3: Teils / teils oder neutral**: Bewertung mit 3, wenn der Befragte der Aussage weder zustimmt noch ablehnt. Dies entspricht einer neutralen Antwort.
- **4: Stimme eher zu**: Bewertung mit 4, wenn der Befragte eher zustimmt, aber nicht vollständig zustimmt.
- **5: Stimme völlig zu**: Bewertung mit 5, wenn der Befragte der Aussage oder Frage stark zustimmt.

**Subskalen-Werte**

Der Helioskope-Fragebogen umfasst drei Subskalen, die unabhängig voneinander zusammengefasst werden können.

Helioskope-Expositionseffekt: Items 2, 6, 7, 9, 10, 12, 14, 18, 19

Helioskope-Schutzeffekt: Items 1, 3, 5, 8, 11, 16, 17, 21

Faktor Vermeidungsstress: Items 4, 13, 15, 20 (maximale Punktzahl 20).

**Gesamtpunktzahl**

Um die Gesamtpunktzahl des Helioskops zu ermitteln, werden die Werte des Schutzeffekts und des Expositionseffekts addiert.

Die maximal mögliche Punktzahl beträgt 85, die niedrigste mögliche Punktzahl 17.

Wenn Sie dieses Kodierungshandbuch befolgen, können Sie den Fragebogen genau bewerten. Wenn Sie weitere Fragen haben oder Hilfe benötigen, wenden Sie sich bitte an

[gregor.hasler@unifr.ch](mailto:gregor.hasler@unifr.ch) oder [vincent.diehl@unifr.ch.](mailto:vincent.diehl@unifr.ch)

## References

Lorenzo-Seva U and Ten Berge JMF (2006) Tucker’s Congruence Coefficient as a Meaningful Index of Factor Similarity. *Methodology* 2(2): 57–64.
